# First Evaluation of PRISMA Level 1 Data for Water Applications

**DOI:** 10.3390/s20164553

**Published:** 2020-08-14

**Authors:** Claudia Giardino, Mariano Bresciani, Federica Braga, Alice Fabbretto, Nicola Ghirardi, Monica Pepe, Marco Gianinetto, Roberto Colombo, Sergio Cogliati, Semhar Ghebrehiwot, Marnix Laanen, Steef Peters, Thomas Schroeder, Javier A. Concha, Vittorio E. Brando

**Affiliations:** 1 Institute for Electromagnetic Sensing of the Environment, National Research Council of Italy (CNR-IREA), 20133 Milan, Italy; bresciani.m@irea.cnr.it (M.B.); alice.fabbretto@gmail.com (A.F.); ghirardi.n@irea.cnr.it (N.G.); pepe.m@irea.cnr.it (M.P.); marco.gianinetto@polimi.it (M.G.); 2 Institute of Marine Sciences—National Research Council (CNR-ISMAR), 30122 Venice, Italy; federica.braga@ismar.cnr.it; 3 Department of Architecture, Built Environment and Construction Engineering, Politecnico di Milano, 20133 Milan, Italy; 4 Remote Sensing of Environmental Dynamics Laboratory, Department of Earth and Environmental Sciences (DISAT), University of Milano-Bicocca, 20126 Milano, Italy; roberto.colombo@unimib.it (R.C.); sergio.cogliati@unimib.it (S.C.); 5 Water Insight, 6709 PG Wageningen, The Netherlands; ghebrehiwot@waterinsight.nl (S.G.); laanen@waterinsight.nl (M.L.); peters@waterinsight.nl (S.P.); 6 CSIRO, Oceans & Atmosphere, Brisbane, QLD 4001, Australia; Thomas.Schroeder@csiro.au; 7 Institute of Marine Sciences, National Research Council of Italy (CNR-ISMAR), 00133 Rome, Italy; Javier.Concha@artov.ismar.cnr.it (J.A.C.); vittorio.brando@cnr.it (V.E.B.)

**Keywords:** imaging spectrometry, inland and coastal waters, fixed position autonomous radiometers

## Abstract

This study presents a first assessment of the Top-Of-Atmosphere (TOA) radiances measured in the visible and near-infrared (VNIR) wavelengths from PRISMA (PRecursore IperSpettrale della Missione Applicativa), the new hyperspectral satellite sensor of the Italian Space Agency in orbit since March 2019. In particular, the radiometrically calibrated PRISMA Level 1 TOA radiances were compared to the TOA radiances simulated with a radiative transfer code, starting from in situ measurements of water reflectance. In situ data were obtained from a set of fixed position autonomous radiometers covering a wide range of water types, encompassing coastal and inland waters. A total of nine match-ups between PRISMA and in situ measurements distributed from July 2019 to June 2020 were analysed. Recognising the role of Sentinel-2 for inland and coastal waters applications, the TOA radiances measured from concurrent Sentinel-2 observations were added to the comparison. The results overall demonstrated that PRISMA VNIR sensor is providing TOA radiances with the same magnitude and shape of those in situ simulated (spectral angle difference, SA, between 0.80 and 3.39; root mean square difference, RMSD, between 0.98 and 4.76 [mW m^−2^ sr^−1^ nm^−1^]), with slightly larger differences at shorter wavelengths. The PRISMA TOA radiances were also found very similar to Sentinel-2 data (RMSD < 3.78 [mW m^−2^ sr^−1^ nm^−1^]), and encourage a synergic use of both sensors for aquatic applications. Further analyses with a higher number of match-ups between PRISMA, in situ and Sentinel-2 data are however recommended to fully characterize the on-orbit calibration of PRISMA for its exploitation in aquatic ecosystem mapping.

## 1. Introduction

Hyperspectral remote sensing techniques for inland and coastal water monitoring have been developed for more than three decades (e.g., [1,2]). Overall, the availability of a continuous spectrum makes algorithms more effective in a wide variety of waters with varying water column depths and bottom reflectance, and lead to a more successful retrieval of a larger number of properties (e.g., [3,4,5,6]). Furthermore, other studies [7,8,9,10] have outlined the need for having a spatial resolution of the order of decametres (hence typical of terrestrial missions like Landsat and Sentinel-2) to capture the fine spatial features typical of inland and coastal waters. Satellite imaging spectrometry might hence offer valuable data to meet such requirements. The Hyperion spectrometer onboard NASA’s Earth Observing One (EO-1) acquired hyperspectral images with a 30-m ground sampling distance (GSD), a 7-km horizontal swath and measuring in the 400–2500 spectral range. From 2001 to 2017, it was used in a variety of studies dealing with inland and coastal waters mapping [11,12,13,14,15]. Since 2001, the Compact High-Resolution Imaging Spectrometer (CHRIS) on ESA’s Proba-1 microsatellite [16] has been providing imagery with a GSD between 18 and 36 m (depending on the operation mode) in the visible and near-infrared (VNIR, 400–1000 nm) spectral region. In the same VNIR range, the Hyperspectral Imager for the Coastal Ocean (HICO) [17] operating onboard the International Space Station (ISS), has been offering imagery with a GSD of 90 m between 2009 and 2015. More recently, the DLR’s Earth Sensing Imaging Spectrometer (DESIS) onboard the ISS is providing very high spectral resolution imagery in the VNIR with a GSD of 30 m [18]. Imagery acquired from CHRIS, HICO and DESIS also provided valuable data for aquatic ecosystems applications (e.g., [19,20,21,22,23]). In 2019, the Italian Space Agency (ASI) launched the PRecursore IperSpettrale della Missione Applicativa (PRISMA) [24]. Other countries have also been developing similar systems, such as the Environmental Mapping and Analysis Program (EnMAP) from Germany, the Hyperspectral Imager Suite (HISUI) from Japan or the Surface Biology and Geology (SBG) from the United States. Details on these spaceborne imaging spectrometry missions are described in [25].

Overall, data gathered from these space missions have supported multiple studies to make progress imaging spectrometry science for inland and coastal waters applications. Nevertheless, being mostly designed for a variety of applications, they might still have some limitations in measuring the generally low signals from water bodies. Compared to other natural surfaces, such as vegetation and soils, the fraction of light reflected from water is in fact very small [26]. Water-leaving radiances are commonly less than 10% of the total radiance measured at Top-Of-Atmosphere (TOA) and are often less than 1% for highly absorbing waters (e.g., those dominated by coloured dissolved organic matter (CDOM)) requiring accurate radiometric characterisation of the sensor [27].

The need for high signal-to-noise ratio (SNR) is actually one of the main drivers in developing satellite missions for ocean colour radiometers, such as the Ocean and Land Colour Instrument (OLCI) onboard of Sentinel-3 [28] or the future NASA Plankton, Aerosol, Cloud, and Ecosystem (PACE) [29]. Nonetheless, sensors mainly designed for land applications (e.g., Landsat-8, Sentinel-2) are often used for inland and coastal waters mapping due to their capacity in observing the aquatic ecosystems at the finer scale (e.g., [30,31,32]). Overall, the development of such applications has been taken with a lot of advantages on studies showing the radiometric characterizations of the sensors for aquatic applications (e.g., [33,34]).

In such a context, this study aims to quantify the on-orbit radiometric quality of PRISMA in the VNIR region. In particular, the TOA radiances obtained from the radiometrically calibrated PRISMA Level 1 data (L1) are compared to the TOA radiances simulated with a radiative transfer code starting from in situ measurements of water reflectance gathered from autonomous radiometers, globally distributed and encompassing five marine locations and a lake. Recognising the role of Sentinel-2 for aquatic applications (e.g., [35,36]), the TOA radiances measured from concurrent observations of Sentinel-2 are included in the analysis to evaluate the match with PRISMA data.

## 2. Materials and Methods

### 2.1. Satellite Data

PRISMA, the ASI sensor launched in orbit by Vega on 22 March 2019, is an innovative system that combines a hyperspectral sensor with a 30-m GSD, with a medium-resolution panchromatic camera with a 5-m GSD (Table 1). In particular, the hyperspectral sensor takes images in a continuum of spectral bands ranging from 400 to 2500 nm. The PRISMA satellite is a single satellite placed in suitable low Earth Sun-synchronous orbit characterized by a repeat cycle of approximately 29 days, with a revisit capability for a specific target of less than one week with off-nadir viewing. It belongs to the class of small satellites, with an expected operating life of 5 years. The PRISMA system provides the capability to acquire, downlink and archive images of all hyperspectral/panchromatic channels totalling 200,000 km^2^ daily over the primary area of interest defined as in the following ranges: 180°W—180°E and 70°S—70°N [37]. A comprehensive description of the PRISMA optical design and technical specifications, including pre-launch and in-flight spectral and radiometric characterization/calibration information, are described in [38]. Notably, the radiometric stability estimated from the onboard calibration unit is better than ±1% for VNIR spectrometer and bandcenter wavelength shift is lower than 0.1 pixel cross-track.

Overall, PRISMA offers the scientific community and users many applications in the field of environmental monitoring and resources management in multiple domains, such as agriculture and forestry, raw material exploration and mining, snow and inland and coastal waters [24,25,26,27,28,29,30,31,32,33,34,35,36,37]. Due to its potential recognised use in aquatic remote sensing [39], PRISMA images are expected to provide significant advance in algorithm development and innovative monitoring tools.

**Figure 1:**
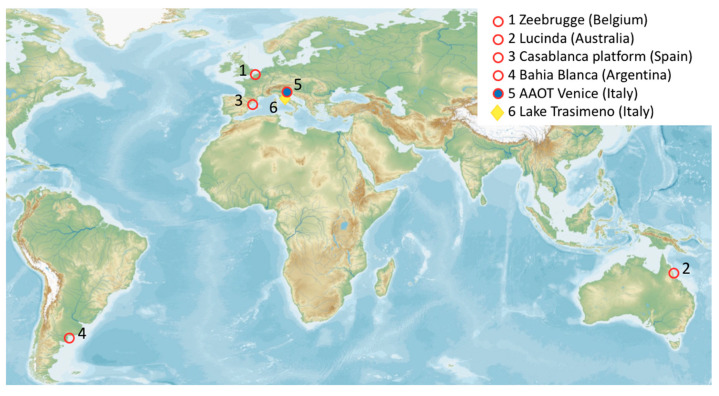
Distribution of in situ dataset: red dots are for AERONET-OC sites, blue dot is for PANTHYR site (colocated with the AERONET-OC AAOT Venice), and yellow diamond is for the WISPStation site.

The PRISMA L1 data products (version 3.6)—distributed in HDF5 file format—were downloaded from the PRISMA portal (https://prisma.asi.it) and re-projected with a geographic lookup table (GLT) Bowtie Correction routine available in the ENVI© software (L3Harris Technologies, Inc., Melbourne, FL, USA) for removing artefacts associated with bowtie effects or missing data. A total of nine cloud-free VNIR scenes acquired from July 2019 to June 2020 were selected. The images, each covering an area of 30 km by 30 km, are distributed rather globally in both hemispheres to cover the sites for which in situ measurements of water reflectance were available during the satellite overpass (cf. Figure 1). The first 57 spectral bands in the 400–900 nm range, with a spectral bandwidth within ~9–12 nm, were extracted from L1 VNIR data to perform the study.

Whenever PRISMA scenes were acquired within +/− 1 day of a corresponding Sentinel-2 overpass, the corresponding L1C Multi Spectral imager (MSI) images were downloaded for a direct comparison with PRISMA. Sentinel-2/MSI images were processed in SNAP toolbox (http://step.esa.int/main/) to transform the dimensionless apparent reflectance into physical units of TOA radiances and for spatially resampling the multispectral cube to 30-m pixel size—hence comparable to the PRISMA GSD. A spectral sub-setting of the first nine bands was also performed as the comparison is limited to the 400–900 nm range.

### 2.2. In Situ Data

Figure 1 shows the geographic distribution of sites (the coordinates are provided in Table 2), providing in situ measures to be compared to PRISMA. The sites represent a rather wide range of water types and trophic conditions but also atmospheric turbidity. In particular: (1) the Zeebrugge site in the North Sea is representative of turbid nearshore waters [40]; (2) the Lucinda site is located in tropical coastal waters in Eastern Australia, dominated by non-algal particulates and CDOM [41,42]; (3) the Casablanca site in the Western Mediterranean Sea is representative of open ocean chlorophyll-a dominated waters [43]; (4) the Bahia Blanca site in the southern Atlantic coast is representative of high suspended loads induced by widespread erosion and strong tidal currents [44]; (5) the Acqua Alta Oceanographic Tower (AAOT) of Venice in the northern Adriatic Sea is representative of moderately sediment dominated waters [45,46]; (6) finally, Lake Trasimeno has shallow turbid waters with recurrent sediment resuspension and moderate to high bloom of phytoplankton, including cyanobacteria species [47].

For each site, in situ measurements of water reflectance were gathered with fixed position autonomous above-water radiometry systems. For marine sites in situ data were provided by AERONET-OC; moreover, for the AERONET-OC AAOT Venice site, in situ data were also collected by a PANTHYR system. Finally, a WISPStation provided in situ measurements for lake Trasimeno, Italy. The next three sections provide details on the automatic systems used in this study.

#### 2.2.1. AERONET-OC System and Network

The AERONET-OC builds on the former AERONET developed to sustain atmospheric studies at various scales with measurements from worldwide distributed autonomous sun-photometers. AERONET-OC has been extended to support aquatic applications by providing the additional capability of measuring the radiance emerging from the water (i.e., water-leaving radiance). These Cimel CE-318 modified sun-photometers, called SeaWiFS Photometer Revision for Incident Surface Measurements (SeaPRISM), have the capability of performing autonomous above-water radiometric measurements in addition to the more common atmospheric measurements. The instruments are typically installed on offshore platforms like lighthouses, oceanographic and oil towers. AERONET-OC is instrumental in satellite ocean colour validation activities through standardized measurements performed at different sites with a single measuring system and protocol, calibrated with an identical reference source and method, and processed with the same code [48,49,50]. In recent years, AERONET-OC has also supported the radiometric characterization of Landsat-8 and Sentinel-2 for aquatic applications [33,34,35,36,37,38,39,40,41,42,43,44,45,46,47,48,49,50,51,52] and, even if for a limited set of bands, we have considered this network very relevant also for PRISMA.

Within the AERONET-OC sites distributed all over the globe, a check was carried out to select the sites which had synchronous measures with PRISMA acquisitions; a total of six match-ups were found as shown in Table 2. The AERONET-OC data collected closer to the sensing time of PRISMA were transformed in water reflectance as described in [52]. Shortly, the upwelling radiance measurements (at multiple wavelengths) made at the AERONET-OC sites were converted to the dimensionless water reflectance above water *r*_w_ (where the dependence on wavelength is omitted in all spectral quantities for the sake of brevity) according to:

*r*_w_ = L_WN_·π/E_0_ (1)

where L_WN_ is the normalized water-leaving radiance downloaded from the AERONET-OC website and the E_0_ is the mean extraterrestrial solar irradiance. The multi-spectral data set obtained from AERONET-OC was also characterised by having six bands common across the six sites.

#### 2.2.2. WISPStation System

The WISPStation is an autonomous radiometer system developed by Water Insight B.V. (Wageningen, The Netherlands). The system is calibrated relative to a reference instrument (calibrated in a certified laboratory using a lamp and an integrating sphere with NIST traceable calibrations) and its usefulness in gathering quality-controlled water reflectance data has been demonstrated over a variety of inland waters [47,48,49,50,51,52,53]. The WISPStation contains two sets of sensors, measuring both the radiance and irradiance near instantaneous at high frequency in the spectral range of 350–900 nm every 15 min. The backend WISPcloud automatically selects the set that is best oriented with respect to the Sun position at any time of the day, eliminating the need for moving parts.

The WISPStation used in this study is located in Lake Trasimeno, a shallow turbid lake of central Italy. The WISPstation, placed on a platform at 400 m from one of the main islands collected remote sensing reflectance (Rrs [sr^−1^]) data every 15 min. WISPStation data overlapping the sensing time of PRISMA within 15 min were averaged. The Rrs measurements from WISPStation were transformed in the dimensionless water reflectance above water *r*_w_ by multiplying Rrs per π.

#### 2.2.3. PANTHYR System

The Pan-And-Tilt HYperspectral Radiometer (PANTHYR) system consists of two TRIOS/RAMSES hyperspectral radiometers, mounted on a pan-and-tilt pointing system, controlled by a single-board-computer and custom-designed electronics which provide power, pointing instructions, and data archiving and transmission [54]. The PANTHYR system acquires high-quality hyperspectral data for water reflectance autonomously every 20 min during daytime over the 350–950 nm spectral range at multiple relative azimuth angles.

An autonomous PANTHYR hyperspectral radiometer was deployed at the AAOT, hence colocated with the AERONET-OC AAOT Venice site. It has been operating since September 2019, and data acquired in coincidence to PRISMA overpasses were used in this study. In particular, although PANTHYR morning measurements are acquired at two relative azimuth angles to the Sun (225° and 270°), data from a single relative azimuth angle (270°) were used in this study [55]. The Rrs measurements from PANTHYR were transformed in the dimensionless water reflectance above water *r*_w_ by multiplying Rrs by π.

### 2.3. Simulation of TOA Radiances

The vector version of the Second Simulation of the Satellite Signal in the Solar Spectrum-vector (6SV) [56,57] was used in this study to simulate the satellite signal starting from in situ measurements of water reflectance. The code has been successfully used for establishing an accurate analytical expression of the reflectance measured by a satellite-sensor that allowed a variety of studies dealing with aquatic applications to be performed (e.g., [58,59,60,61]). Briefly, the TOA radiances to be compared with PRISMA L1 data were simulated using in situ reflectance, by assuming a uniform Lambertian surface and horizontally homogeneous atmospheric layers in a cloudless atmosphere according to the following 6SV scheme:

(2) $$L_{\mathrm{TOA}}=\frac{r_{w}\cdot E_{on-ground}\cdot \tau_{↑}}{\pi \left(1-S\bar{\rho }\right)}+L_{path}$$

where:

- *r_w_* is the water reflectance above water;
- $L_{\mathrm{TOA}}$ is the TOA radiance at sensor level;
- $\tau_{↑}$ is the atmospheric transmittance in the upwelling path;
- $S\bar{\rho }$ is the contribution of the surrounding reflectance (*S* is the spherical albedo);
- $L_{path}$ is the radiance scattered by the atmosphere into the sensor’s field of view before ever reaching the water body;
- $E_{on-ground}$ is the spectral irradiance at ground level which is composed by two components, direct and diffuse irradiance:

(3) $$E_{on-ground}=E_{↓}+E_{0}\cos\left(\theta_{SZ}\right)\tau_{↓}$$

where:

- $E_{↓}$ is the spectral irradiance from the Sun to the Earth’s surface due to the contribution of Rayleigh and Mie scattering;
- $E_{0}$ is the extraterrestrial solar irradiance;
- $\theta_{SZ}$ is the Sun zenith angle;
- $\tau_{↓}$ is the atmospheric transmittance in the downwelling path;

The 6SV code was run with a basic set of input data such as geometrical conditions (i.e., altitude of the target -different from zero for Lake Trasimeno only, Sun azimuth and Sun zenith angles at the time of PRISMA overpass, viewing angles of PRISMA), an atmospheric model for gaseous components according to the latitude and the season, aerosol model type (maritime for the AERONET-OC sites and rural for Lake Trasimeno), the aerosol optical depth (AOD) at 550 nm and water vapour concentrations gathered from AERONET (in case of Trasimeno data from the closer station were used), and the water reflectance *r_w_* from in situ measurements (i.e., the quantity to be simulated to the TOA) with the corresponding spectral resolution.

### 2.4. Match-Up Analysis

Table 2 shows an overview of match-ups used to evaluate PRISMA with respect to both in situ simulated and Sentinel-2 data. Along with the geographic coordinates of the sites (cf. Figure 1), the table indicates the time of in situ measurements for the 6SV simulations with corresponding solar zenith angles, AOD at 550 nm (at PRISMA sensing time) and view zenith angles of PRISMA. Sentinel-2 images acquired on the same day of PRISMA, or within +/− 1 day difference, are also listed in Table 2, along with sensing times. Sentinel-2 data to be compared to PRISMA were available for all sites, save for Lucinda.

For the match-up analysis, the TOA radiances are derived from imagery data by computing the average value and standard deviation of PRISMA and MSI data corresponding to a square box of 3 × 3 pixels defined over the in situ measurements stations. Common descriptive statistical metrics (e.g., [62]) such as root mean square difference (RMSD), mean absolute difference (MAD), spectral angle (SA), and the square of the coefficient of correlation (R^2^) (Table 3) and scatterplots are used for the comparison. A good agreement between the datasets is achieved when R^2^ is close to 1, and bias and the other parameters are close to 0.

For an initial assessment, TOA radiances of PRISMA, in situ simulated and Sentinel-2 MSI data are qualitatively compared at their original spectral resolutions in graphs showing the TOA radiances depending on wavelengths from 400 to 900 nm. Scatterplots and descriptive statistics (Table 3) were then used to quantitatively evaluate the agreement between PRISMA and 6SV simulations for the corresponding bands. To the aim, the higher spectral resolution WISPStation and PANTHYR data were spectrally resampled according to the full width half maximum (FWHM) of L1 PRISMA products; in case of AERONET-OC, the in situ simulated spectra were assumed directly comparable to PRISMA as both sensors have ~10 nm bandwidths. The comparison was carried out both as image-by-image, to provide an evaluation for every single scene (across all bands) and band-by-band, to evaluate a set of specific bands common to both PRISMA and AERONET-OC (across all scenes). Finally, scatterplots and descriptive statistics (Table 3) were also adopted to compare, band-by-band, PRISMA and Sentinel-2/MSI data. To the aim, the spectral response function of MSI filters was used for spectrally resampling the PRISMA TOA radiances.

## 3. Results and Discussion

**Figure 2:**
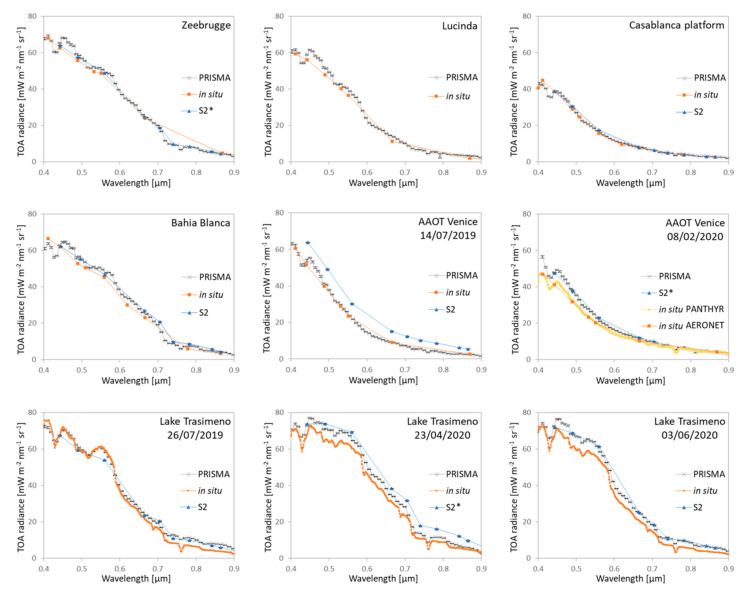
TOA radiances measured by PRISMA and Sentinel-2/MSI (S2), and simulated with 6SV (labelled as in situ). Symbol * means that Sentinel-2 and PRISMA overpasses occurred on the same day.

Figure 2 shows the comparison between the PRISMA, Sentinel-2/MSI and in situ simulated TOA radiances. The data are plotted at their original spectral resolution, and thus Sentinel-2/MSI data provides a smaller number of spectral channels than PRISMA; this is also valid for simulated TOA radiances obtained from AERONET-OC, while the simulated TOA radiances from WISPStation and PANTHYR hyperspectral data provide more spectral information for the comparison. Overall, PRISMA matches well with both 6SV simulations and Sentinel-2/MSI measures. On the marine sites, the best matches between PRISMA, 6SV simulations and Sentinel-2/MSI are obtained in Zeebrugge and Casablanca test sites, which represent two rather different conditions in terms of signal: higher signal for Zeebrugge site (imagery data acquired with SZA of about 30°) and lower signal for Casablanca site (imagery data acquired with SZA of about 60°). For AAOT Venice with reference data acquired on 14 July 2019, Sentinel-2/MSI data was higher than both PRISMA and 6SV simulations; this was due to presence of Sun glint in the MSI image acquired the day before. In the case of Lake Trasimeno, the TOA radiances from PRISMA, Sentinel-2/MSI and in situ simulated look similar both in magnitude and shape. The 6SV simulation obtained from WISPStation and PANTHYR data show, similar to PRISMA, the features of atmospheric gaseous absorptions (e.g., H_2_O) as also observed from other space borne hyperspectral sensors [11,63].

**Figure 3:**
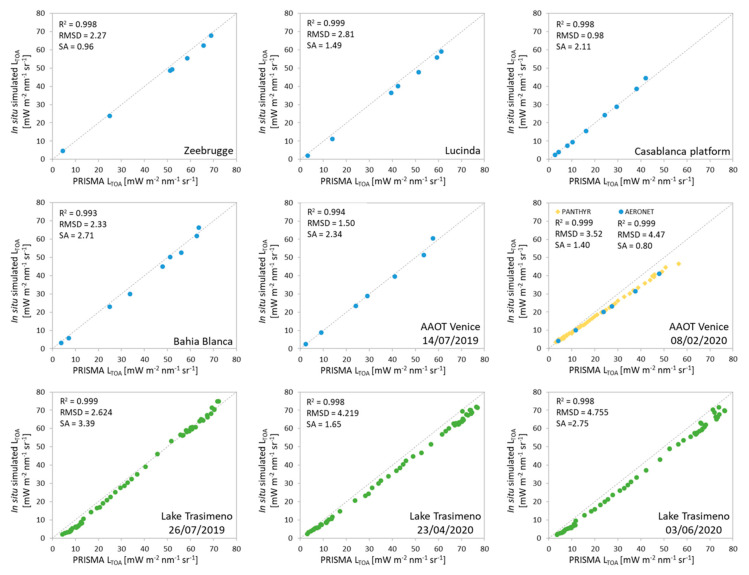
Scatterplots of TOA radiances from PRISMA (x-axis) vs. 6SV simulations (y-axis) for common bands. Blue dots are indicating the comparison for AERONET-OC sites, green for WISPStation and yellow for PANTHYR.

The image-by-image comparison of PRISMA with 6SV simulations (Figure 3) shows a R^2^ of about 0.99 indicating a strong linear correlation in the VNIR range. RMSDs is between 0.98 [mW m^−2^ sr^−1^ nm^−1^] for the Casablanca platform site and 4.76 [mW m^−2^ sr^−1^ nm^−1^] for the Lake Trasimeno site (03/06/2020) hence indicating an overall good correspondence between the two set of data. Metrics also show a good agreement in terms of spectral shape, with SA ranging from 0.96 [°] for the Zeebrugge to 2.71 [°] for Bahia Blanca with simulated data obtained from AERONET-OC. In AAOT Venice site (08/02/2020) the results obtained with PANTHYR are comparable to the outcomes of AERONET-OC, even if some difference is present due to the different characteristics of data set such as the number of bands available to compute the metrics.

**Figure 4:**
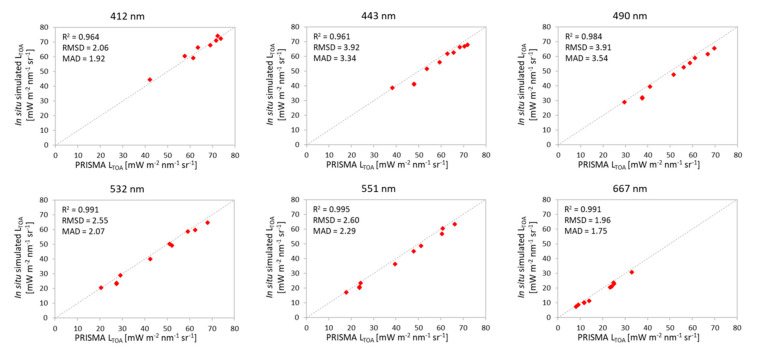
Scatterplots of TOA radiances from PRISMA (x-axis) vs. 6SV simulations from in situ data (y-axis) and PRISMA (x-axis) for the nine test sites. The plots are generated for wavelengths (in nm) of the six bands which are common to all in situ data.

**Figure 5:**
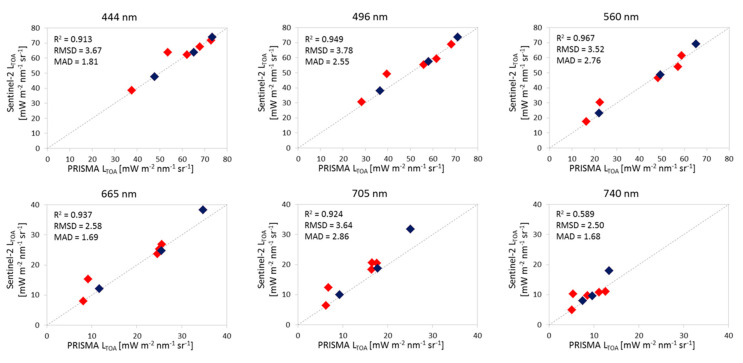
Scatterplots of TOA radiances from PRISMA (x-axis) vs. Sentinel-2/MSI (y-axis) for the eight match-ups (corresponding to five sites, i.e., all save Lucinda). Diamonds in blue highlight when PRISMA and Sentinel-2/MSI were acquired on the same day, diamonds in red stand for +/− 1 day mismatch.

Figure 4 shows the outcomes of the band-by-band analysis. PRISMA and the 6SV simulation shows a high degree of fitting with R^2^ values higher than 0.9 for all the six bands. The RMSDs are in the range of 1.96–3.92 [mW m^−2^ sr^−1^ nm^−1^], with slightly higher RMSDs for bands at 443 nm and 490 nm. A similar comparison but for PRISMA with Sentinel-2/MSI is shown in Figure 5. When resampling PRISMA bands to Sentinel-2/MSI, TOA radiances measured by both satellite sensors are very similar. No differences are appreciable for images acquired on the same day or within +/− 1 day difference, which is also indicating a low tidal influence or stability of water masses. Data appears slightly more scattered at longest wavelengths (R^2^ = 0.589 at 740 nm) probably due to the lower range of variation of TOA radiances observable over water.

Overall, the results on the good agreement of PRISMA TOA radiances with respect to both in situ simulated data and Sentinel-2 images are indicating the potentialities of PRISMA in observing water targets. Existing sources of uncertainties due to the execution of the 6SV without to account for neither adjacency effects or the sky radiance at the air/water interface were also not evidently captured from the analysis of the present dataset. However, for a more comprehensive study on PRISMA, these effects should be considered as respectively suggested by [64] and [65]. The number of match-ups also seems limited to revealing differences attributable to different illumination or viewing geometries as observed in other studies where discrepancies in TOA radiances measured by different sensors were explained by different viewing and solar zenith angles [34]. Apart from a difference due to Sun glint for one of the nine match-ups, the good agreement between PRISMA and Sentinel-2 confirms how imaging spectrometry and Sentinel-2 might be combined for developing water resource applications [66].

## 4. Conclusions

This study reports a first comparison of TOA radiances within the VNIR range from PRISMA L1 products and 6SV simulations using simultaneous in situ measurements. A total of nine PRISMA scenes acquired from July 2019 to June 2020 over different water targets across the globe were investigated. The in situ radiometric measurements were used as input to the 6SV radiative transfer code, which simulated the expected TOA radiances with a physical-based approach consistent with PRISMA observations (i.e., viewing geometry, Sun position, aerosol optical thickness at the time of PRISMA overpass). Moreover, the corresponding Sentinel-2/MSI acquired within +/− 1-day of PRISMA overpasses were added to the analysis for all sites except for Lucinda. The comparisons between PRISMA and 6SV simulations overall show a good agreement; similarly, for PRISMA and Sentinel2/MSI, which also showed a high degree of fit.

This study demonstrated that PRISMA TOA radiances are consistent with the expected values observable over water targets hence encouraging the exploitation of imagery acquired by PRISMA mission for aquatic applications. Since PRISMA TOA radiances are comparable to Sentinel-2/MSI, and seeing that Sentinel-2 provides a five-day revisit time, their synergic use seems also very promising for aquatic remote sensing as outlined in [66]. The use of fixed position autonomous radiometers, whose advantages have been largely demonstrated in various studies (e.g., [33,49,61]), was revealed to be fundamental to performing this first analysis during times of limited fieldwork and research voyage opportunities due to the global COVID-19 pandemic.

Although the results are promising, further analyses are necessary to fully characterize the on-orbit calibration of PRISMA for water applications. To this aim, further efforts on the use of more match-ups between satellite and in situ measurements, as well as an improvement of the radiative transfer simulation are required. Such an exercise might help to evaluate PRISMA radiometry with respect to measurement requirements for observing aquatic ecosystems [67] and to contribute to the definition of vicarious calibration gains specific for water. Further work is also needed to investigate both the SNR and PRISMA L2 products, which were not considered in this study, but that are critical for developing aquatic applications and studies (e.g., [33,68]).

## Figures and Tables

**Table 1:** Main features of the PRISMA payload.

**Orbit altitude reference**	615 km
**Field of View (FOV)**	2.77°
**Instantaneous FOV**	48.34 mrad
**Swath**	30 km
**Ground Sampling Distance**	Hyperspectral camera: 30 m
	Panchromatic camera: 5 m
**Spectral range**	VNIR: 400–1010 nm (66 bands)
	SWIR: 920–2500 nm (173 bands)
	PAN: 400–700 nm
**Signal-to-noise ratio**	VNIR: >160 (450 at 650 nm)
	SWIR: >100 (>360 at 1550 nm)
	PAN: >240
**Spectral Width**	≤14 nm
**Spectral Calibration Accuracy**	±0.1 nm
**Radiometric quantisation**	12 bits
**Repeat cycle**	29 days (450 orbits)
**Relook time**	<7 days
**Lifetime**	5 years

**Table 2:** Site names; coordinates (in decimal degrees) of in situ AERONET-OC, PANTHYR and WISPStation sensors with the time (hh.mm) of the measures used in the 6SV simulation (in case of WISPStation an average value in the defined range); aerosol optical depth at 550 nm (AOD) and Sun zenith angle (SZA in degree) at the sensing time of PRISMA; view zenith angle (VZA in degree) of PRISMA; dates and sensing times of PRISMA and Sentinel-2.

Site Name	Lat, Long and Time of In Situ Data Gathering	AOD	SZA	VZA	PRISMA	Sentinel-2
Zeebrugge	51.362 N, 3.120 E; AERONET-OC 10:28	0.15	33.15	1.62	23 July 2019 10:57:56	23 July 2019 10:56:29
Lucinda	18.520 S, 146.386 E; AERONET-OC 01:57	0.04	36.61	15	25 July 2019 00:30:40	no overpass
Casablanca platform	40.717 N, 1.358 E; AERONET-OC 11:34	0.12	60.66	5	26 December 2019 10:51:57	27 December 2019 10:53:49
Bahia Blanca	39.148 S, 61.722 W; AERONET-OC 13:06	0.06	41.59	5	27 February 2020 14:14:31	26 February 2020 14:00:49
AAOT Venice	45.314 N, 12.508 E; AERONET-OC 10:21	0.097	27.56	15	14 July 2019 10:05:58	13 July 2019 10:10:31
AAOT Venice	45.314 N, 12.508 E; AERONET-OC 09:36, PANTHYR 10:00-10:20	0.24	62.80	5	8 February 2020 10:10:29	8 February 2020 10:11:51
Lake Trasimeno	43.121 N, 12.130 E; WISPStation 10:00-10:15	0.18	27.30	0.8	26 July 2019 10:13:20	25 July 2019 10:00:39
Lake Trasimeno	43.121 N, 12.130 E; WISPStation 10:00-10:15	0.11	33.47	15	23 April 2020 10:04:28	23 April 2020 10:05:49
Lake Trasimeno	43.121 N, 12.130 E; WISPStation 10:00-10:15	0.13	24.03	5	03 June 2020 10:10:59	02 June 2020 10:05:59

**Table 3:** Statistical metrics used to assess the agreement of TOA radiances among the datasets; n is the number of concurrent observations of the match-up, x is for PRISMA, y is for in situ simulated or Sentinel-2.

Statistical Metric	Equation
Root Mean Square Difference (RMSD)	$\mathrm{RMSD}=\sqrt{\frac{\sum_{i=1}^{n}{\left(y_{i}-x_{i}\right)}^{2}}{n}}$
Mean Absolute Difference (MAD)	$\mathrm{MAD}=\frac{\sum_{i=1}^{n}\left|y_{i}-x_{i}\right|}{n}$
Spectral Angle (SA)	$\mathrm{SA}=\cos^{-1}\frac{\sum_{i=1}^{n}y_{i}x_{i}}{\sqrt{\sum_{i=1}^{n}y_{i}^{2}}\sqrt{\sum_{i=1}^{n}x_{i}^{2}}}$
Square of the coefficient of correlation (R^2^)	$R^{2}=\frac{\sum_{i=1}^{n}\left(x_{i}-\bar{x}\right)\left(y_{i}-\bar{y}\right)}{\sqrt{\sum_{i=1}^{n}{\left(x_{i}-\bar{x}\right)}^{2}}\sqrt{\sum_{i=1}^{n}{\left(y_{i}-\bar{y}\right)}^{2}}}$
